# Association of High Serum Levels of Growth Factors with Good Outcome in Ischemic Stroke: a Multicenter Study

**DOI:** 10.1007/s12975-019-00747-2

**Published:** 2019-11-25

**Authors:** Tomás Sobrino, Manuel Rodríguez-Yáñez, Francisco Campos, Ramón Iglesias-Rey, Mónica Millán, Natalia Pérez de la Ossa, Antonio Dávalos, Raquel Delgado-Mederos, Alejandro Martínez-Domeño, Joan Martí-Fábregas, Mar Castellanos, Joaquín Serena, Aida Lago, Exuperio Díez-Tejedor, José Castillo

**Affiliations:** 1 Clinical Neurosciences Research Laboratory, Health Research Institute of Santiago de Compostela (IDIS), Santiago de Compostela, Spain; 2 Department of Neurosciences - Acute Stroke Unit, Hospital Universitari Germans Trias i Pujol, Universidad Autònoma de Barcelona, Badalona, Spain; 3 Stroke Unit, Neurology Department, Hospital de la Santa Creu i Sant Pau, Barcelona, Spain; 4 Department of Neurology - Stroke Unit, Biomedical Research Institute of Girona, Hospital Universitario Doctor Josep Trueta, Girona, Spain; 5 Department of Neurology, Complexo Hospitalario Universitario da Coruña, A Coruña, Spain; 6 Department of Neurology, Hospital Universitario La Fe, Valencia, Spain; 7 Department of Neurology and Stroke Center, Neurosciences Area, IdiPAZ (Health Research Institute), La Paz University Hospital, Autónoma University of Madrid, Madrid, Spain

**Keywords:** Growth factors, Infarct volume, Ischemic stroke, Prognosis

## Abstract

The main objective of this research work was to study the association of serum levels of growth factors (GF) and SDF-1α with the functional outcome and reduction of lesion volume in ischemic stroke patients. In this multicenter study, 552 patients with non-lacunar stroke (male, 62.1%; mean age, 68.2 ± 11.4) were included within 24 h from symptom onset. The main outcome variable was good functional outcome (modified Rankin Scale [mRS] ≤ 2) at 12 months. Secondary outcome variable was infarct volume (in mL) after 6 ± 3 months. Serum levels of VEGF, Ang-1, G-CSF, BDNF, and SDF-1α were measured by ELISA at admission, 7 ± 1 days, at 3 ± 1 months, and 12 ± 3 months. Except for BDNF, all GF and SDF-1α serum levels showed a peak value at day 7 and remained elevated during the first 3 months (all *p* < 0.01). High serum levels at day 7 of VEGF (OR, 19.3), Ang-1 (OR, 14.7), G-CSF (OR, 9.6), and SDF-1α (OR, 28.5) were independently associated with good outcome at 12 months (all *p* < 0.0001). On the other hand, serum levels of VEGF (B, − 21.4), G-CSF (B, − 14.0), Ang-1 (B, − 13.3), and SDF-1α (B, − 44.6) measured at day 7 were independently associated with lesion volume at 6 months (*p* < 0.01). In summary, high serum levels of VEGF, Ang-1, G-CSF, and SDF-1α at day 7 and 3 months after ischemic stroke are associated with good functional outcome and smaller residual lesion at 1 year of follow-up.

## Introduction

Improvement of the management of stroke patients, especially in developed countries, has contributed to the reduction in mortality and morbidity rates observed in recent decades. However, the incidence of stroke has not followed this same trend showing a continuous increase in developed countries [1]. Besides, the demographic change expected in Europe for the next 50 years suggests that this situation will become worse [2].

In addition, the ceiling of reperfusion treatment is already close (only 20% of with ischemic stroke patients can benefit from these treatments in highly specialized hospitals, whose and their effectiveness does not exceed 50–60%) [3]. Moreover, new neuroprotective approaches are limited, mainly due to a narrow therapeutic window. Therefore, the identification of new targets for ischemic stroke is mandatory for clinicians and researchers.

The medium-long-term prognosis (weeks and/or months) of patients with ischemic stroke will depend on the size and topography of the infarction, the speed and efficiency of the acute phase therapeutic measures, and the degree of neurorepair processes mediated by the patient’s cerebral plasticity mechanisms [4, 5].

One of the main options for enhancing the mechanisms of cellular plasticity is cellular therapy that, favors by the extraordinary advance of the investigation of cellular biology, represents a hopeful therapeutic target for ischemic stroke. In this regard, the use of embryonic stem cells, induced pluripotent stem cells, and adult stem cells have demonstrated therapeutic effects in preclinical models of cerebral ischemia, and to a lesser extent in humans mainly through the mesenchymal stem cells. However, the mechanisms underlying cellular therapy are not well understood, since most of the transplanted cells disappear within a few weeks, and this therapy is not free from potentially serious complications [6–8]. It is possible that effectiveness of cellular therapy is based on trophic factors and other bioactive substances that modify the cerebral microenvironment and stimulate the development of the patient’s own cellular niches [9–11]. Therefore, the stimulation of cerebral plasticity by using growth factors (GF), which participate in a coadjutant way in neurogenesis, neoangiogenesis, gliagenesis, and synaptogenesis mechanisms, may constitute a more translational therapeutic approach from the lab to the bedside [12, 13].

Unlike the great existing knowledge on GF in preclinical models of cerebral ischemia, there is a gap in studies in clinical practice which may be one of the reasons why clinical trials with GF such as granulocyte colony-stimulating factor (G-CSF) have failed [13, 14]. Thus, it is necessary to know better the relationship between growth factors and the prognosis of ischemic stroke patients at long term.

For this purpose, we designed a multicenter and observational study of a cohort of patients with acute ischemic stroke in order to study the association of serum levels of GF (vascular endothelial growth factor [VEGF], [G-CSF], brain-derived neurotrophic factor [BDNF], angiopoietin 1 [Ang-1]) and stromal-derived factor-1α [SDF-1α] with the functional outcome and reduction of lesion volume of ischemic stroke patients during a follow-up period of 1 year.

## Materials and Methods

### Study Population and Patient Characteristics

We have conducted a multicenter and observational study of a cohort of ischemic stroke patients, of less than 24 h from stroke onset, consecutively admitted to Stroke Units of 6 Spanish hospitals. The period of recruitment of the patients was carried out in the first half of 2014. Patients without diagnostic confirmation by neuroimaging, lacunar infarcts, previous disability (modified Rankin Scale > 1), cancer, severe systemic or metabolic disease, and inflammatory or infectious disease in the previous 15 days and patients who received investigational drugs in clinical trials were excluded.

### Clinical Variables

All patients were admitted to an acute Stroke Unit and treated according to the European Stroke Organisation guidelines [15]. The follow-up period was 1 year, including 2 visits at 3 ± 1 months and at 12 ± 3 months.

Medical history recording potential vascular risk factors, blood and coagulation test, 12-lead electrocardiogram, chest X-ray, and carotid ultrasonography were performed at admission. Stroke subtype was classified according to the TOAST criteria [16], and stroke severity was assessed by a certified neurologist using the National Institute of Health Stroke Scale (NIHSS) at admission, 48 h, 3 ± 1, and 12 ± 3 months. Functional outcome was evaluated at 3 ± 1 and 12 ± 3 months using the modified Rankin Scale (mRS). Good functional outcome was defined as a mRS score ≤ 2. Physiotherapy/rehabilitation was collected during the hospitalization as well as the first 3 or 12 months of follow-up period.

### Neuroimaging Variables

Cerebral computed tomography (CT) studies were carried out at admission, between 4th and 7th days and after 3 months. Patients who received reperfusion treatment had another CT study before and at 24–36 h after treatment. Hemorrhagic transformation (HT) was assessed in the follow-up CT. HT was defined as symptomatic when it was associated with early neurological deterioration (worsening > 4 points in the NIHSS during the first 48 h from stroke onset). Lesion volume was calculated on the follow-up CT by using the formula 0.5 × A × B × C, where A and B are the largest perpendicular diameters, and C is the number of 1-cm thick sections that contain the lesion. All neuroimaging evaluations were made by investigators who had no knowledge of the patients’ clinical and laboratory results.

### Outcome Variables

The primary endpoint was good functional outcome (mRS score ≤ 2) at 12 ± 3 months; as secondary variables, we considered the good functional outcome (mRS score ≤ 2) at 3 ± 1 months and the lesion volumes between 4th and 7th days and after 3 months.

### Laboratory Tests

Biochemistry, hematology, and coagulation test were assessed in the central laboratory of each participating hospital. However, the growth factors and SDF-1α determinations of the total patients included in each of 6 hospitals were performed in the Clinical University Hospital of Santiago de Compostela. The GF selected for this study were VEGF, G-CSF, BDNF, Ang-1, and SDF-1α. For these molecular determinations, venous blood samples were collected in Vacutainer tubes (Becton Dickinson, San Jose, CA, USA) at admission, 7 ± 1 days, 3 ± 1, and 12 ± 3 months. After allowing to clot for 60 min, blood samples were centrifuged at 3000×*g* for 10 min, and the serum was immediately aliquoted, frozen, and stored at − 80 °C until analysis. Serum levels of VEGF, G-CSF, and BDNF (Boster Biological Technology, Encyclopedia Circle Fremont, CA, USA), Ang-1 (Adipo Bioscience Inc., Santa Clara, CA, USA), and SDF-1α (R&D Systems Inc., Minneapolis, MN, USA) were quantified using commercial ELISA kits following instructions provided by commercial houses. The coefficients of intra-assay variation were VEGF, 4.4%; G-CSF, 5.2%; BDNF, 4.8%; Ang-1, 4.7%; and SDF-1α, 3.6%; the coefficients of inter-assay variation were VEGF, 6.9%; G-CSF, 8.3%; BDNF, 7.9%; Ang-1, 8.1%; and SDF-1α, 7.2%. The determinations were performed in an independent laboratory that did not have access to clinical or neuroimaging data.

### Statistical Analysis

According to unpublished own previous results, 42% (p1 = 0.42) of ischemic stroke patients have a mRS > 2 at discharge and do not show a significant improvement during a 1 year of follow-up period. It has been calculated that if a sample of 545 individuals (*n* = 545) is included, an accuracy of 4.12% (ω = 0.41) will be obtained by means of a normal 95% asymptotic confidence interval (*γ* = 0.95) bilateral (*c* = 2). For the calculation of the sample size, EPIDAT version 4.2 (www.sergas.es/Saude-publica/EPIDAT) was used.

Results were expressed as percentages for categorical variables and as mean (SD) or median and interquartile range for the continuous variables depending on their distribution. Kolmogorov-Smirnov test was used for testing normality. Chi-square or Fisher test was performed to study categorical variables. The continuous were studied with the Student’s *t* or the Mann-Whitney tests. Spearman’s or Pearson’s analyses were used to test bivariate correlations. ANOVA was used to analyze the relationship between stroke subtypes and growth factors. Mixed analysis of variance (MANOVA) was used to study the effect of time, and groups (good and poor outcome) by time (baseline to 12 months) interactions on GF and SDF-1α levels. The influence of growth factors on functional outcome and the lesion volume was assessed by logistic regression and multiple lineal regression models, respectively. The models were constructed considering the baseline variables related to each outcome variable. Results were expressed as adjusted odds ratios (OR) or Beta estimate with their 95% confidence intervals (95% CI). The statistical analysis was conducted using IBM SPSS Statistic, version 20 (SPSS Inc. Chicago, IL, USA).

## Results

### Sample Description

A total of 650 patients with a first-ever ischemic stroke of less than 24 h from symptoms onset was included in the study. Six patients were excluded due to lack of diagnostic confirmation, 39 patients were lacunar infarcts, 4 patients showed previous disability (mRS > 1), and 12 patients were included in clinical trials during the acute phase. Moreover, 37 patients were loss during follow-up or suffered a recurrent stroke. Therefore, a final sample of 552 patients (male, 62.1%; mean age, 68.2 ± 11.4 years) was valid for testing the primary endpoint of the study.

**Fig. 1:**
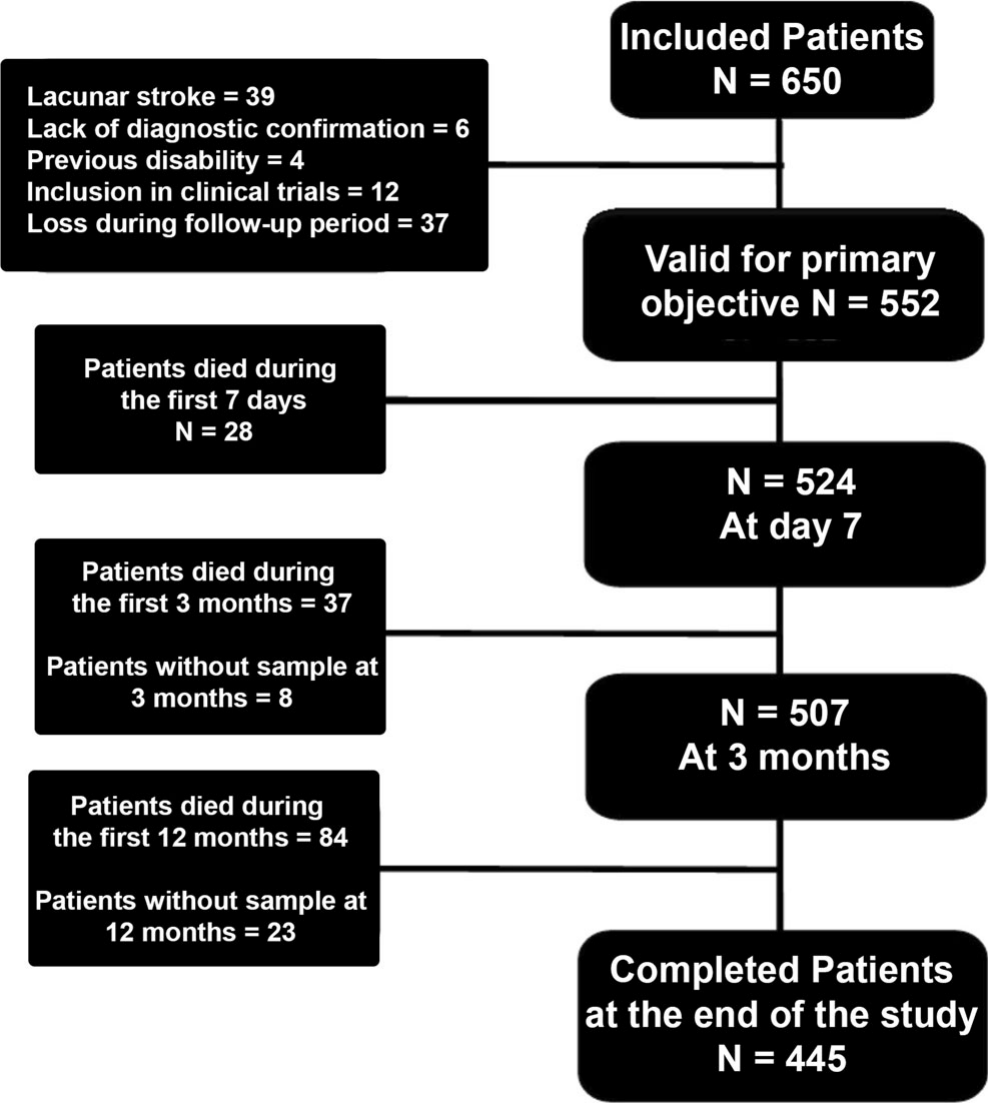
Screening and enrollment into the study

On the other hand, 28 patients died during the first month/week/admission, 37 at 3 months, and 84 at the end of the follow-up period of 12 months. Additionally, blood samples were not obtained in 8 patients at 3 months, and 23 patients had not blood sample at 1 year of follow-up. So, a baseline serum sample was available in 552 patients, at 7 days in 524, at 3 months in 507, and finally 445 patients had serum sample at 12 months (Fig. 1).

Regarding neuroimaging, CT at admission was performed in all; the second CT performed between 4th and 7th days was available in 537 patients, while the final CT was completed in 409 patients after 3 months (average time, 6.3 ± 2.7 months after admission).

NIHSS at admission was 11 [6, 17]. A total of 198 patients (35.9%) received intravenous fibrinolytic treatment within the first 4.5 h. Following TOATS criteria, 13.9% of patients were diagnosed as atherothrombotic stroke, 50.0% cardioembolic, and 36.1% as undetermined origin. The median (quartiles) of mRS at 3 months was 2 [1, 3] and 1 [0, 3] at 12 months.

### Temporal Profile of GF and SDF-1α Levels

**Fig. 2:**
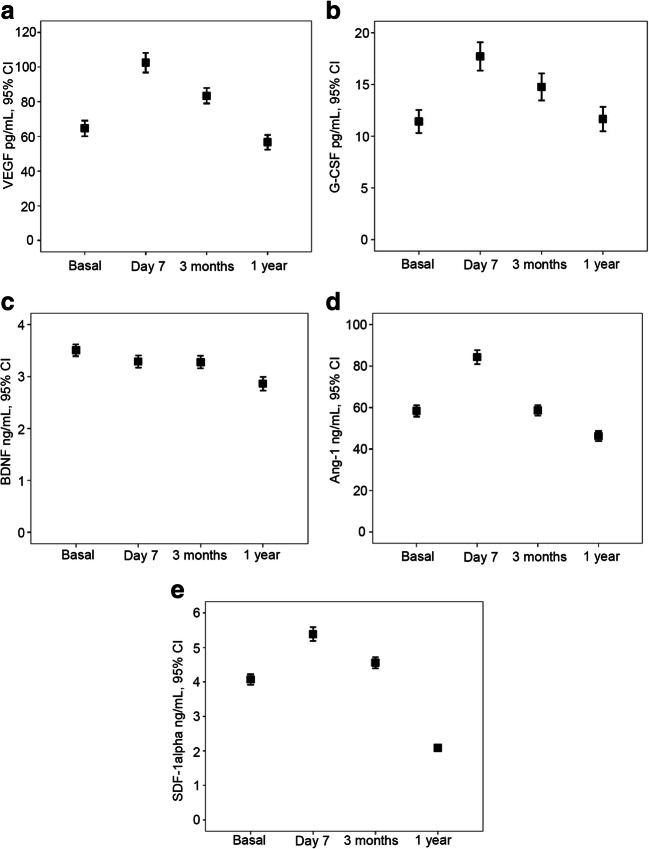
(**a–e**) Temporal profile of GF (VEGF, G-CSF, BDNF, Ang-1) and SDF-1α levels at admission, 7 ± 1 days, 3 ± 1 months, and 12 ± 3 months. All GF and SDF-1α serum levels showed a peak value at day 7 and remained elevated during the first 3 months (all *p* < 0.01), except for BDNF

Except for BDNF, all GF and SDF-1α serum levels showed a peak value at day 7 (all *p* < 0.01) (Fig. 2). The increase in percentage of serum levels of GF during the first week were as follows: VEGF, 77.4 ± 111.8% (*p* < 0.0001); G-CSF, 67.2 ± 98.8% (*p* < 0.0001); BDNF, − 2.1 ± 44.3% (*p* = 0.349); Ang-1, 69.6 ± 120.6% (*p* < 0.0001); and SDF-1α, 29.6 ± 31.4% (*p* < 0.0001). At 3 months, only an increase of VEGF (*p* < 0.0001) and G-CSF (*p* < 0.0001) serum levels in relation to the basal concentration persisted; the concentrations of BDNF (*p* = 0.312), Ang-1 (*p* = 0.632), and SDF-1α (*p* = 0.078) were similar to baseline. By contrast, at 12 months, serum levels of VEGF (*p* = 0.169) and G-CSF (*p* = 0.418) were similar to those at baseline, but BDNF (*p* < 0.0001), Ang-1 (*p* = 0.016), and SDF-1α (*p* < 0.0001) levels were lower.

### Primary Outcome: Good Functional Outcome at 12 Months

Table 1 shows the main characteristics of patients by functional outcome groups. Three hundred and fifty-one (68.6%) patients showed good functional outcome at 12 months. Patients with good functional outcome were younger, showed milder stroke severity, and smaller lesion volume at 4th–7th day. Furthermore, fibrinolytic treatment, rehabilitation, and statins treatment previous and during acute phase of stroke were more frequent in the group of patients with good outcome.

**Table 1:** Baseline clinical characteristics, vascular risk factors, stroke subtype, biochemical parameters and neuroimaging findings in patients with good or poor outcome at 12 ± 3 months

	Good outcome *n* = 351	Poor outcome *n* = 201	*p* value
Age, years	66.9 ± 11.6	69.6 ± 9.4	0.001
Males, %	61.3	63.7	0.586
History of hypertension, %	58.4	71.6	0.002
History of diabetes, %	19.4	27.9	0.026
Smoking habit, %	26.8	13.4	< 0.0001
Alcohol consumption, %	15.4	9.5	0.051
History of hyperlipidemia, %	41.3	55.2	0.002
History of ischemic heart disease, %	15.4	16.9	0.631
History of atrial fibrillation, %	24.8	34.8	0.014
History of heart failure, %	10.3	9.5	0.883
Previous disability (mRS < 1)	0 (0, 0)	0 (0, 0)	0.448
Statins prior to stroke, %	31.3	21.1	0.008
Axillary temperature admission, °C	36.4 ± 0.5	36.6 ± 0.5	0.080
Glucose levels, mg/dL	127.6 ± 50.4	143.2 ± 43.5	< 0.0001
Leukocytes, × 10^3^/mmc	9.3 ± 6.1	9.1 ± 2.4	0.100
Fibrinogen, mg/dL	404.7 ± 133.4	439.6 ± 160.1	0.003
NIHSS at admission	8 (5, 13)	18 (14, 20)	< 0.0001
Fibrinolytic treatment, %	39.0	30.3	0.043
Infarct volume (4th to 7th day), mL	16.8 ± 29.8	98.2 ± 94.1	< 0.0001
Hemorrhagic transformation, %	13.4	16.9	0.263
Statins after stroke, %	70.9	62.2	0.038
TOAST			0.113
Atherothrombotic, %	11.7	17.9	
Cardioembolic, %	48.1	53.2	
Indeterminate, %	40.2	28.9	
Rehabilitation during-hospital, %	27.1	33.0	0.145
Rehabilitation during 12 months, %	10.3	18.9	0.007

**Fig. 3:**
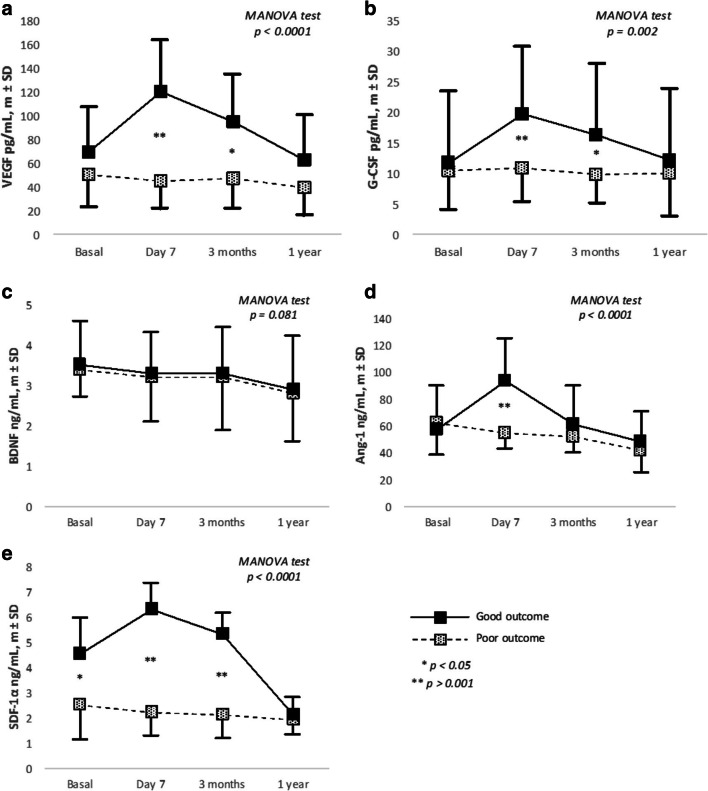
(**a–e**) Temporal profile serum levels of GF (VEGF, G-CSF, BDNF, Ang-1) and SDF-1α in patients with good or poor outcome at 12 ± 3 months

On the other hand, with the exception of BDNF, all GF and SDF-1α showed a different expression profile in patients with good and poor outcome. Mean (SD) levels of VEGF, G-CSF, and SDF-α at day 7 and 3 months as well as Ang-1 at day 7 were significantly higher in patients with good functional outcome (Fig. 3).

In the logistic regression analysis, serum levels of SDF-1α (OR, 28.5; 95% CI 13.2–81.2), VEGF (OR, 19.3; 95% CI 10.7–231.9), Ang-1 (OR, 14.7; 95% CI 4.4–61.9), and G-CSF (OR, 9.6, 95% CI 6.9–55.3) at day 7 were independently associated with good functional outcome at 12 months after adjustment by significant variables of bivariate analysis showed in Table 1. Moreover, serum levels of SDF-1α (OR, 20.2; 95% CI 8.1–57.1), VEGF (OR, 10.9; 95% CI 2.3–50.7), and G-CSF (OR, 8.9; 95% CI 4.4–34.7) at 3 months were also independently associated with good functional outcome at 12 months (Table 2).

**Table 2:** Adjusted and non-adjusted OR of good outcome at 12 months for serum levels of GF (VEGF, G-CSF, BDNF, Ang-1) and SDF-1α at admission, day 7 ± 1, 3 ± 1 months, and 12 ± 3 months

Growth factors	Non-adjusted			Adjusted*		
	OR	95% CI	*p*	OR	95% CI	*p* value
VEGF at admission	1.7	1.3–2.3	0.001	1.7	1.5–4.9	0.001
VEGF at 7 days	26.6	14.1–48.7	< 0.0001	19.3	10.7–231.9	< 0.0001
VEGF at 3 months	22.3	11.7–42.5	< 0.0001	10.9	2.3–50.7	< 0.0001
VEGF at 12 months	3.1	2.0–4.9	< 0.0001	1.6	1.1–10.0	< 0.0001
G-CSF at admission	0.8	0.5–1.1	0.255	0.85	0.4–1.5	0.603
G-CSF at 7 days	15.9	8.8–28.9	< 0.0001	9.59	6.9–55.3	< 0.0001
G-CSF at 3 months	6.5	3.7–11.4	< 0.0001	8.99	4.4–34.7	< 0.0001
G-CSF at 12 months	1.4	0.9–2.3	0.124	1.11	0.5–2.3	0.781
BDNF at admission	1.3	0.7–2.1	0.305	0.69	0.2–1.9	0.494
BDNF at 7 days	2.2	1.4–3.6	0.001	2.69	0.9–6.9	0.139
BDNF at 3 months	1.9	1.1–3.1	0.017	1.48	0.5–3.6	0.395
BDNF at 12 months	1.3	0.7–2.4	0.337	1.75	0.5–5.1	0.308
Ang-1 at admission	0.8	0.5–1.2	0.279	1.47	0.7–2.7	0.230
Ang-1 at 7 days	29.6	15.4–57.1	< 0.0001	14.72	4.4–61.9	< 0.0001
Ang-1 at 3 months	1.5	0.9–2.2	0.062	1.57	0.7–3.4	0.251
Ang-1 at 12 months	1.3	0.8–1.9	0.293	1.26	0.6–2.6	0.536
SDF-1α at admission	22.1	10.1–126.8	< 0.0001	16.25	10.1–87.2	< 0.0001
SDF-1α at 7 days	31.9	15.8–214.1	< 0.0001	28.53	13.2–81.1	< 0.0001
SDF-1α at 3 months	29.6	10.3–103.8	< 0.0001	20.19	8.1–57.2	< 0.0001
SDF-1α at 12 months	2.3	1.0–5.3	0.043	2.09	0.6–7.8	0.269

*Adjusted by age, history of hypertension, history of diabetes, smoking habit, history of hyperlipidemia, history of atrial fibrillation, statins prior to stroke, glucose levels, fibrinogen, NIHSS at admission, fibrinolytic treatment, infarc volume, rehabilitation during 12 months

### Secondary Outcome: Good Functional Outcome at 3 Months

Three hundred and forty-seven patients (62.9%) had good functional outcome at 3 months. Bivariate analysis showed similar results to those obtained for the functional prognosis at 12 months (data not shown). The logistic regression analysis showed that serum levels of SDF-1α (OR, 20.6; 95% CI 9.2–46.1), VEGF (OR, 12.6; 95% CI 5.7–27.7), Ang-1 (OR, 9.1; 95% CI 4.0–20.8), and G-CSF (OR, 6.2; 95% CI 3.2–21.3) at day 7 were independently associated with good functional outcome at 3 months.

### Secondary Outcome: Lesion Volume at 4th–7th Days and at 6 Months

**Fig. 4:**
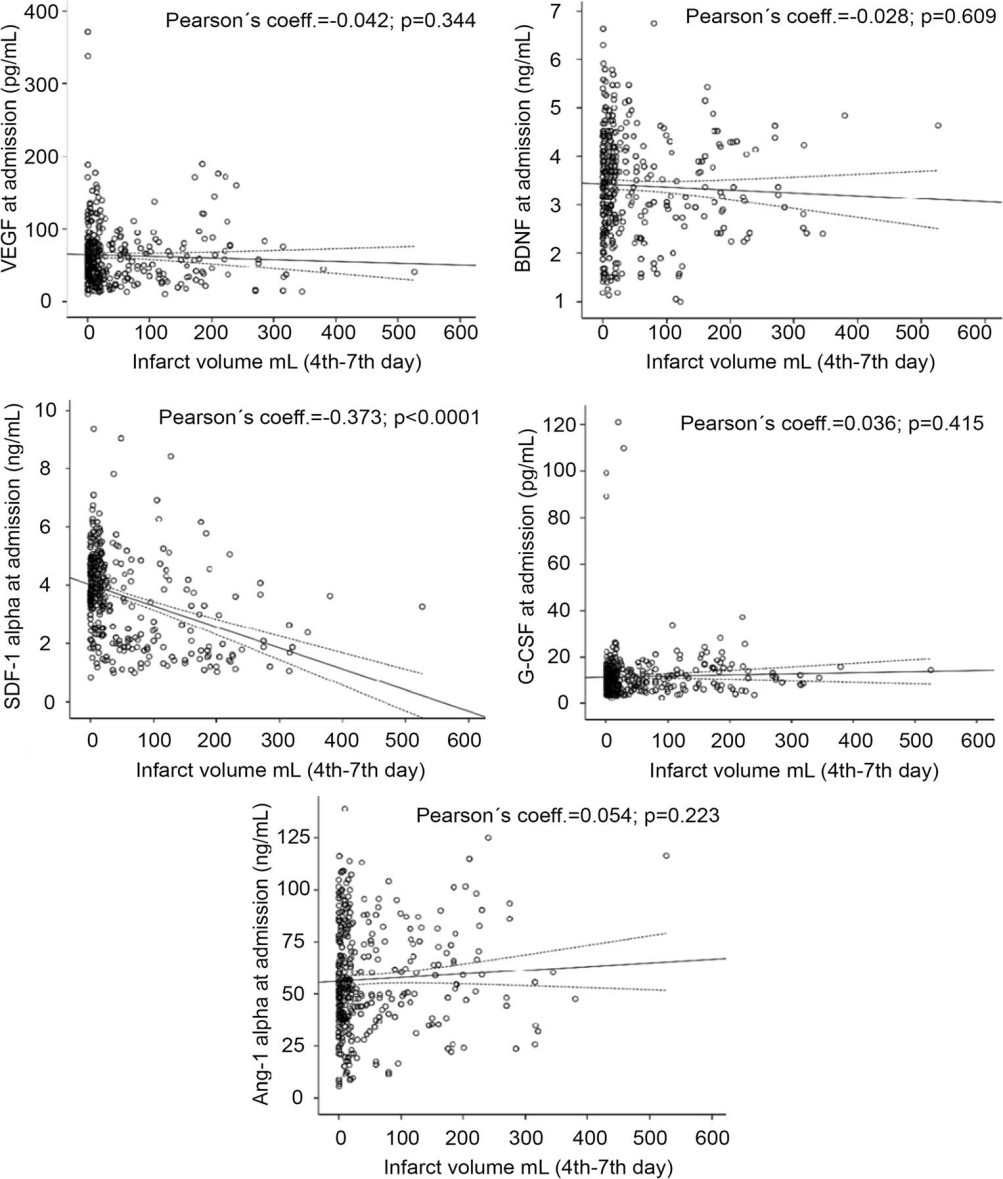
Scatterplots between GF (VEGF, G-CSF, BDNF, Ang-1) and SDF-1α serum levels measured at admission and 7 ± 1 days and the lesion volumes at 4th–7th day

**Fig. 5:**
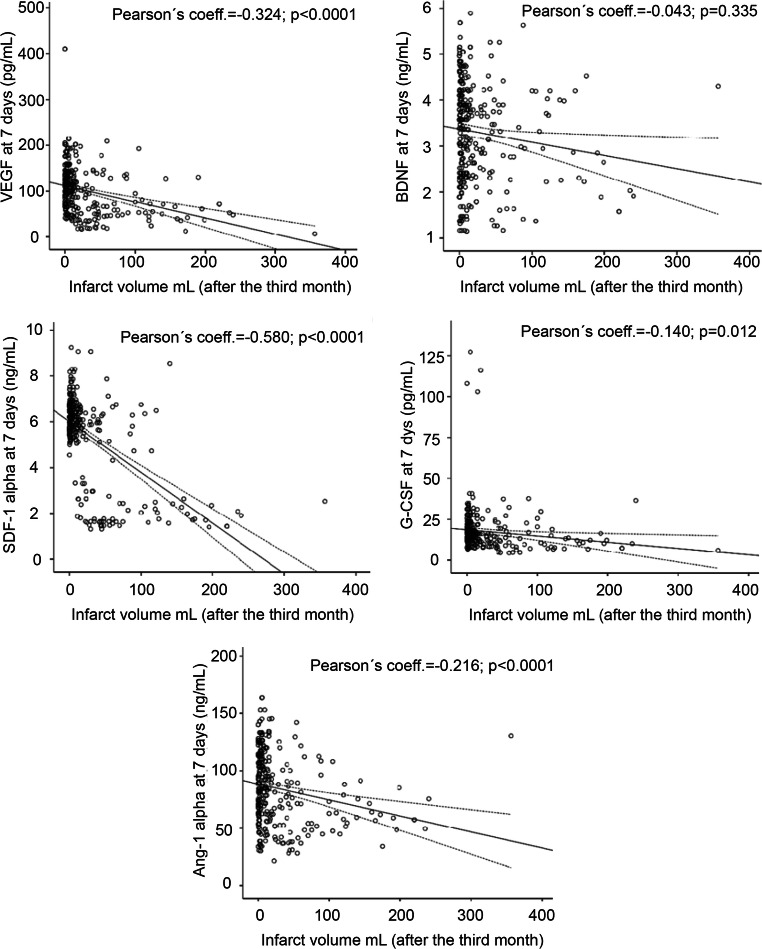
Scatterplots between GF (VEGF, G-CSF, BDNF, Ang-1) and SDF-1α serum levels measured at 7 ± 1 days and after 6 ± 3 months

Figures 4 and 5 show the correlations between GF and SDF-1α serum levels, measured at admission and the lesion volumes at days 4–7, the correlation between GF and SDF-1α serum levels at day 7, and lesion volume at 6 months. Only admission serum levels of SDF-1α (*r* = − 0.373) were associated with lesion volume at 4th–7th day. However, serum levels at day 7 of VEGF (*r* = − 0.324), G-CSF (*r* = − 0.140), Ang-1 (*r* = − 0.216), and SDF-1α (*r* = − 0.580), but not BDNF, had a significant correlation with the lesion volume after 3 months.

In the multiple linear regression model, adjusted for those significant variables associated to lesion volume in the univariate analysis (history of arterial hypertension, alcohol consumption, history of atrial fibrillation, fibrinolytic treatment, and NIHSS at admission), serum levels of VEGF (B, − 21.4), G-CSF (B, − 14.0), Ang-1 (B, − 13.3), and SDF-1α (B, − 44.6) measured at day 7 were independently associated with lesion volume at 6 months (all *p* < 0.01). By contrast, only SDF-1α levels (B, − 29.6) at admission were independently associated to lesion volume at 4th–7th day (Table 3).

**Table 3:** Adjusted* B of lesion volume at 4th–7th day and after 3 months for serum levels of GF (VEGF, G-CSF, BDNF, Ang-1) and SDF-1α at admission and day 7 ± 1

Admission	Lesion volume (4th–7th day)			7th day	Infarct volume (at 6 month)		
	*B*	95% CI	*p* value		*B*	95% CI	*p* value
VEGF	− 4.9	− 13.6 to 3.79	0.269	VEGF	− 21.6	− 29.6 to − 3.7	< 0.0001
G-CSF	− 1.4	− 11.1 to 21.42	0.125	G-CSF	− 14.0	− 23.6 to − 4.7	0.003
BDNF	3.5	− 11.6 to 18.56	0.650	BDNF	− 11.2	− 23.4 to 1.1	0.074
Ang-1	− 3.3	− 13.60 to 7.01	0.530	Ang-1	− 13.3	− 24.5 to − 2.0	0.021
SDF-1α	− 29.2	− 41.95 to − 16.50	< 0.0001	SDF-1α	− 44.6	− 54.2 to − 34.9	< 0.0001

*Adjusted by age, history of atrial fibrillation, NIHSS at admission, and, fibrinolytic treatment

## Discussion

This prospective and multicenter study shows a relationship between serum GF and SDF-1α and brain injury in patients with ischemic stroke. The increase of serum levels of VEGF, Ang-1, G-CSF, and SDF-1α at day 7 and 3 months after ischemic stroke was independently associated with a good functional outcome at 3 and 12 months. This favorable effect on the primary variable was supported by positive effects on the reduction of lesion volume after 3 months. Importantly, these effects remained significant after adjustment for well-recognized prognostic factors such as age, lesion volume, stroke severity, and fibrinolytic treatment.

Serum levels of VEGF, G-CSF, Ang-1, and SDF-1α showed a similar biological pattern, with a peak value at day 7 and remained elevated levels during the first 3 months. Of all of them, SDF-1α was the biomarker more powerful associated to the outcome variables (functional recovery and residual lesion volume). Conversely, BDNF had a different profile, showing a progressive decrease of its serum levels from the stroke onset. In fact, BDNF levels were not associated with any of the endpoints analyzed in this study, neither with de infarct volume, nor with the functional outcome during a follow-up period of 1 year. The role of BDNF in the pathophysiology of ischemic stroke has been evaluated in animal and clinical studies, where the results were not conclusive. Previous studies [17, 18] have presented a relationship between a baseline and isolated BDNF determination and outcome of ischemic stroke patients (in our series there was a slight non-significant association with a lower infarct volume); moreover, the increase of the BDNF expression after rehabilitation has also been reported [19]. Other studies supported the lack of correlation between serum BDNF levels and lesion size or recovery in stroke patients [20, 21]. However, it is important to note that different research works have showed that serum BDNF may not accurately reflect BDNF concentrations in the brain due to endothelial dysfunction [22, 23]. BDNF levels could be also influenced by a variety of factors including platelet count, gender, smoking status, depression, and age and Val66Met polymorphism [21, 24–28]. Therefore, further studies are necessary to elucidate the potential role of BDNF as a GF able to induce recovery in ischemic stroke patients.

The selection of the GF analyzed in this study was performed due to previous studies, mainly in preclinical models of cerebral ischemia, since there is a gap in studies in clinical practice which may be one of the reasons why clinical trials with GF such as G-CSF have failed [13, 14]. Specifically, VEGF is a potent angiogenic factor, inducible by hypoxia, which plays an important role in the vascular response to ischemia, promoting the formation of new cerebral blood vessels (neovascularization) [29–34]. Likewise, VEGF has been also implicated as a factor promoting neurogenesis in the adult brain, increasing the proliferation and differentiation of endothelial and neuronal progenitor cells [9, 29, 35].

G-CSF induces the mobilization of hematopoietic stem cells from the bone marrow to the injured brain regions, reducing the volume of the infarct and improving neuronal plasticity and vascularization, which translates into a better functional recovery [36–38]. Also inhibits apoptosis and stimulates the differentiation of neuronal progenitor cells, inducing neurogenesis.

BDNF is a neurotropic factor involved in ischemia-induced neurogenesis processes, and increased recruitment of endogenous progenitors to injured brain regions, mediating repair mechanisms and neuronal plasticity [39]. BDNF has also been shown to induce synaptogenesis, morphogenesis, and plasticity of dendritic spines, resulting in synapses with functionality [40, 41].

The functionality of GF depends, to a large extent, on the relationship between them and between the cells that induce their expression. Thus, VEGF induces the release of BDNF from endothelial cells [42] and stimulates the formation of pericytes [31]. Similarly, Ang-1 and SDF-1α could be the link between neurogenesis and angiogenesis, promote neuroblast migration, and post-stroke functional recovery [43, 44].

The usefulness of the knowledge about the potential role of GF in the recovery of the cerebral lesion caused by the ischemia is not conditioned only by a better understanding of the underlying mechanisms that mediate brain repair but also because they may imply more translational therapies than those mediated by cellular therapy. In this regard, drugs that induce the expression of GF are already known, such as 4α-phorbol 12,13-didecanoate (an endothelial receptor agonist), 15(S)-hydroxyeicosat acid (arachidonic acid metabolite) [34], or guanosine [45]. In addition, GF have already been tested in clinical trials, although with modest results. The G-CSF has shown a slight functional improvement, but not of the neurological deficit [14].

This study has some limitations. First, patients were admitted to Stroke Units of 6 Spanish hospitals, and hematology and coagulation test were assessed in the central laboratory of each participating hospital. However, patients were treated according to the European Stroke Organisation guidelines [15]. In addition, the growth factors and SDF-1α determinations of the total patients were performed only in one hospital. Second, to have normal healthy subjects as a control group (cohort of age, comorbidity) would give more value to the results presented. Three, it is important to note that many conditions affect the level of growth factors, for example, subject exercise or age [21, 24–28]. Although given the mean age of patients (68.2 ± 11.4 years), their physical activity or exercise was low or moderate. Four, we did not take the location of the lesion into account in our analysis, only the lesion volume. Five, we have not considered in the analysis the different reperfusion treatments (intravenous or intraarterial fibrinolysis, thrombectomy, or both procedures). Six, the design of our study does not allow concluding mechanistic aspects on the role of growth factors in the prognosis of patients with cerebral infarction. However, its results open the possibility to design new more translational clinical trials. Short-term administrations of growth factors during the acute or subacute phases of stroke are not expected to modify the prognosis of patients. According to the temporal expression profiles of GF obtained, an administration started within the first week and prolonged during the first 3 months after the stroke seems a more translational approach. However, for this objective, new therapeutic formulations will be necessary [13].

## Summary

High serum levels of VEGF, Ang-1, G-CSF, and SDF-1α at day 7 and 3 months after ischemic stroke are associated with good functional outcome and smaller residual lesion at 1 year of follow-up. New clinical trials, not focused in acute phase of stroke, should be conducted in order to clarify whether GF may be able to promote functional recovery in ischemic stroke patients.
